# Safety profile of biologic drugs for psoriasis in clinical practice: An Italian prospective pharmacovigilance study

**DOI:** 10.1371/journal.pone.0241575

**Published:** 2020-11-03

**Authors:** Luigi Francesco Iannone, Luigi Bennardo, Caterina Palleria, Roberta Roberti, Caterina De Sarro, Maria Diana Naturale, Stefano Dastoli, Luca Donato, Antonia Manti, Giancarlo Valenti, Domenico D’Amico, Santo D’Attola, Adele Emanuela De Francesco, Vincenzo Bosco, Eugenio Donato Di Paola, Steven Paul Nisticò, Rita Citraro, Emilio Russo, Giovambattista De Sarro

**Affiliations:** 1 Science of Health Department, School of Medicine, University Magna Graecia, Catanzaro, Italy; 2 Dermatology Outpatient Clinic, Azienda Sanitaria Provinciale Crotone, Crotone, Italy; 3 Division of Dermatology, "Ciaccio-Pugliese" Hospital, Catanzaro, Italy; 4 Dermatologic Unit, Grande Ospedale Metropolitano “Bianchi-Melacrino-Morelli”, Reggio Calabria, Italy; 5 UOC Pharmacy, “Mater domini” Hospital, Catanzaro, Italy; Humanitas University, ITALY

## Abstract

Psoriasis is an inflammatory and chronic skin disorder associated with physical and psychological burden impairing patients’ quality of life. In the last decade, biologic drugs have widely changed treatment of moderate-severe psoriasis and their number is increasing overtime. To early identify expected/unexpected adverse events (AEs) with biologic treatments, pharmacovigilance programs are needed. We designed a post-marketing active pharmacovigilance program to monitor and analyse AEs and/or serious adverse events (SAEs) reports. All consecutive patients treated with one biologic drug during a two-years period and satisfying inclusion criteria have been enrolled in five Dermatology tertiary units. Demographic and clinical features of patients, type of treatment used, therapy discontinuation, failures, switch/swap to another biologic, and possible onset of AEs were collected. Overall, 512 patients with a diagnosis of psoriasis (286; 55.9%) or arthropathic psoriasis (226; 44.1%) have been enrolled. Eighty-two (16%) patients with AEs and 5 (1%) with SAEs have been identified. Further, 59 (11.5%) had a primary/secondary failure (mainly on infliximab and etanercept). The adverse events and SAEs were reported with golimumab (4/12), adalimumab (32/167), infliximab (9/48), etanercept (31/175) and ustekinumab (11/73), no adverse events have occurred with secukinumab (0/37). Infliximab and etanercept were significantly associated with primary/secondary failures, whereas no differences have been highlighted for AEs insurgence. On the other hand, ustekinumab seems to be associated with a low rate of AEs (p = 0.01) and no adverse events or failures have been reported with secukinumab (p = 0.04 and 0.03, respectively). Our study, even though limited by *a small sample size and a brief follow-up period*, provide useful data on widely used biologic drugs and their tolerability, discontinuation rate and the incurrence of severe adverse events. Further studies are necessary to include the recently approved biologic drugs and to increase the sample size for more detailed analysis.

## Introduction

Psoriasis is an inflammatory and chronic skin disorder affecting about 3% of the population worldwide, associated with physical and psychological burden impairing patients’ quality of life [1].

Compared to general population, several chronic diseases have a higher incidence in patients with psoriasis such as psoriatic arthritis (10–30% prevalence), cardiovascular disorders, Crohn’s disease and depression, increasing both morbidity and mortality [2,3].

Nowadays, according to evidence-based guidelines [4,5], treatments for psoriasis include topical therapy, phototherapy, conventional and biological systemic treatments.

In Europe, for moderate to severe psoriasis, systemic drugs are the treatments of choice subdivided in a first line of conventional drugs (methotrexate, cyclosporine A and retinoids) and a second line of biologic drugs approved for psoriasis and psoriatic arthritis (TNF-α inhibitors [infliximab, etanercept, adalimumab], IL-12 and 23 blockers [ustekinumab and guselkumab] and anti-IL17 [secukinumab, ixekizumab and brodalumab]) or for arthropathic psoriasis (golimumab, a TNF-α inhibitor) [4].

Over the past 15 years, biologic therapies have revolutionized the treatment of moderate-severe psoriasis with substantial improvements in patients’ management. Biologic drugs have several advantages compared to conventional treatment including no evidence of cumulative toxicity or clinical-relevant drug-drug interactions, established long-term efficacy and warranted use in renal or hepatic impaired patients [6]. However, rare and unpredictable adverse events are difficult to detect in pre-marketing clinical trials due to their inclusion criteria and small sample size. Moreover, biologic drugs can be associated to adverse events (AEs) not related to their specific mechanism of action but by triggering unwanted immune response, with anti-drugs antibodies production [7].

Phase IV studies can provide data from spontaneous reporting systems on long term efficacy, tolerability and evidencing potential predictors of ineffectiveness, AEs and/or severe AEs (SAEs) insurgence [8].

Nevertheless, surveillance on adverse events on biologic drugs, as well as other drug classes, is far from being optimal and underreporting is still pervasive, and frequently the spontaneous reporting of AEs underestimates the real number of events [9,10]. Active pharmacovigilance activities may be more useful for the detection and reporting of serious and unexpected AEs [8,11] although some limitations must be considered [12].

Aim of this active post-marketing study was to monitor and analyse AEs occurring with biologic drugs using the data from the dermatologic area of the active Calabria Biologics Pharmacovigilance Program [9].

## Materials and methods

### Study design and data collection

The Calabria Biologics Pharmacovigilance Program (CBPP) is a multicentre pharmacovigilance study as previously described [9,13]. Briefly, CBPP is a multicenter pharmacovigilance study aimed at improving the continuous monitoring of safety of treatment with biologic drugs in clinical practice. Furthermore, the program provides regular training sessions to physicians on pharmacovigilance reports and AEs accurate identification. Data have been obtained during two years of the CBPP for the evaluation of safety and appropriateness of biologics prescription in dermatologic units. All consecutive patients undergoing treatment with one biologic drug at 5 tertiary centres (Dermatology Outpatient clinics of *Azienda Ospedaliera “Pugliese-Ciaccio”*, Catanzaro, Italy; *Azienda Ospedaliera Provinciale Crotone*, Crotone, Italy; *Grande Ospedale Metropolitano* “Bianchi-Melacrino-Morelli”, Reggio Calabria, Italy; A*zienda Ospedaliera* “Mater Domini”, Catanzaro, Italy; *Azienda Ospedaliera Cosenza*, Cosenza Italy) between January 1, 2016 and December 31, 2017, have been screened for study eligibility.

All the patients enrolled in the study have entirely met the following inclusion criteria: age ≥ 18 years; diagnosis of moderate to severe psoriasis (Ps) or arthropathic psoriasis (PsA) and treatment with one biologic drug. The “*index date*” has been identified at the beginning of the first biologic treatment during the study protocol for each patient (naïve or from a previous biologic drug). The follow-up period, started at the index date, has been characterized by a hybrid AEs detecting system (phone calls by pharmacologist as above described and routine specialist visits).

The following data have been collected from each enrolled patient: demographic and clinical characteristics such as age, sex, diagnosis, disease duration, current or prior use of biologic drugs, corticosteroids, other treatments, relevant comorbidities, possible discontinuation or switch/swap to another drug with motivation, potential primary or secondary failure, and AEs onset.

Patients were considered to have discontinued treatment if they had not taken biologic therapy within the recommended time or if they had not renewed their therapeutic plan. Reasons for treatment discontinuation (withdraw with or without therapy switch) in our study included primary/secondary failure (*i*.*e*. no response to a new biologic drug administered or fails to respond within 16 weeks) or development of AE. Furthermore, patients experienced AEs or therapeutic failures have been subdivided in subgroups and compared to patients without adverse events or inefficacy to treatment to highlight significative differences.

For each AE reported, the onset date, severity, time-course, duration and outcome have been descripted and coded according to the MedDRA dictionary version 20.0 and drugs’ SmPC and *EudraVigilance* have been checked to assess previous reports.

Furthermore, in order to evaluate the association (causal relationship) between AEs and drug treatment, the Naranjo Adverse Probability Scale [14] was applied. For each AE reported, (excluded site injection AEs, clearly linked to administration), a clinical pharmacologist validated the causal link using the Naranjo algorithm and assigned a score to classify AE in certain (>8) probable (5–8) possible (1–4) doubtful (0).

An AE was defined as serious if was life-threatening or fatal, required hospitalization (or prolonged existing hospitalization), resulted in persistent or significant disability or in a congenital anomaly/birth defect or was another medically important condition (European Medicines Agency, 2017). Drugs’ SmPC and *EudraVigilance* have been checked to assess previous reports.

Moreover, to compare the number of AEs spontaneously reported for biologics in the same tertiary centres 24 months before starting the protocol, AEs in the “*Rete Nazionale Farmacovigilanza*” (RNF) have been reported.

Written informed consent was obtained from all patients and were informed that medical records will be anonymously utilized for studies. The study protocol was approved by the local Ethics Committee (*Comitato Etico Regionale Calabria*, Italy), protocol number 278/2015. All procedures were performed in accordance with the 1964 Declaration of Helsinki and its later amendments.

### Statistical analysis

Sample size was based on patient’s enrolment on each study site and not precalculated. A descriptive analysis was executed to summarize basal and demographic characteristics of enrolled patients at index date. Continuous data are presented as mean ± standard deviation (SD) or median (25–75 percentile) as appropriate, while categorical data are expressed as number (percentage). Considering that the Kolmogorov-Smirnov test has noted that some of the numerical variables were not normally distributed, a non-parametric approach was applied. In detail, the Mann–Whitney U test for continuous variables and two-tailed Pearson chi-squared test or Fisher’s test for categorical variables were used to compare data.

Odds ratios (ORs) and 95% confidence intervals (CIs) were calculated using univariate and multivariate regression models to evaluate the contribution of independent variables in predicting adverse events and primary/secondary failure.The significance was set at a *p* value < 0.05. SPSS 26.0 software (Chicago, IL) was used for statistical and data analysis.

## Results

### General characteristics of the study population

Overall, 512 patients (204 females; mean age 54.9 ± 13.1 years) with a diagnosis of active Ps (286, 55.9%) or PsA (226, 44.1%) started a treatment with a biologic drug and have been enrolled. All demographic and clinical information are summarized in Table 1.

**Table 1:** Characteristics of the study cohort.

	Overall patients (n = 512)
**Age**, years	54.9 ± 13.1
**Female sex**, n (%)	204 (39.8)
**Follow up**, months	19 ± 1.5
**Age first biologic therapy**, years	51.3 ± 13.6
**Naïve,** n (%)	394 (77.0)
*Diagnosis*	
**Plaque psoriasis**, n (%)	286 (55.9)
**Psoriatic arthritis**, n (%)	226 (44.1)
*Biologic drugs prescribed*	
**IFX**, n (%)	48 (9.4)
**ETN**, n (%)	175 (34.2)
**ADA**, n (%)	167 (32.6)
**GOL**, n (%)	12 (2.3)
**UST**, n (%)	73 (14.3)
**SEC**, n (%)	37 (7.2)
*Concurrent treatments*	104 (20.3)
**MTX**, n (%)	52 (10.2)
**CyA**, n (%)	76 (14.8)
**Acitetrin**, n (%)	5 (1.0)
**CCS**, n (%)	2 (0.4)
**NSAIDs**, n (%)	0
**PUVA**, n (%)	1 (0.2)
**Apremilast**, n (%)	4 (0.8)
**Switched**, n (%)	103 (20.1)
*Adverse events*	
**AEs**, n (%)	82 (16.0)
**SAEs**, n (%)	5 (1.0)

IFX, infliximab; ETN, etanercept; ADA, adalimumab; GOL, golimumab UST, ustekinumab; SEC, secukinumab; MTX, methotrexate; CyA, cyclosporin A; CCS, corticosteroids; NSAIDs, Nonsteroidal anti-inflammatory drugs; PUVA, Psoralen Ultra-Violet A; AEs, adverse events; SAEs, serious adverse events.

Etanercept (ETN) was the most commonly administered biologic drug at the index date (175; 34.2%), followed by adalimumab (ADA) (167; 32.6%), ustekinumab (UST) (73; 14.3%), infliximab (IFX) (48; 9.2%), secukinumab (SEC) (37; 7.2%) and golimumab (GOL) (12, 2.3%). Data subdivided *per* drugs are reported in Table 2.

**Table 2:** Characteristics of the study cohort *per* drugs.

	IFX (n = 48)	ETN (n = 175)	ADA (n = 167)	GOL (n = 12)	UST (n = 73)	SEC (n = 37)
**Age**, years	55.1 ± 12.6	55.8 ± 12.6	55.6 ± 13.1	50.2 ± 13.2	52.6 ± 13.7	54.2 ± 14.6
**Male sex**, n (%)	27 (56.2)	111 (63.4)	92 (55.1)	7 (58.3)	46 (63.0)	26 (70.3)
**Follow up**, months	18.4 ± 1.5	20 ± 1.3	19.1 ± 2.5	18 ± 0.5	21 ± 1	18.4 ± 2
**Age first biologic therapy**, years	50.8 ± 13.6	50.7 ± 12.9	53.3 ± 13.7	48.2 ± 13.9	50.0 ± 14.5	52.5 ± 14.6
**Naïve,** n (%)	31 (64.6)	116 (66.3)	137 (82.0)	12 (100)	63 (86.3)	35 (94.6)
*Diagnosis*						
**Plaque psoriasis**, n (%)	23 (47.9)	82 (46.9)	92 (55.1)	0	53 (72.6)	35 (94.6)
**Psoriatic arthritis**, n (%)	25 (52.1)	93 (53.1)	75 (44.9)	12 (100)	20 (27.4)	2 (5.4)
*Concurrent treatments*	20 (41.7)	37 (21.1)	32 (19.2)	4 (33.3)	9 (12.3)	3 (8.1)
**MTX**, n (%)	15 (31.2)	15 (8.6)	17 (9.7)	3 (25)	1 (1.4)	1 (2.7)
**CyA**, n (%)	15 (31.2)	29 (16.6)	21 (12.6)	1 (8.3)	7 (9.6)	3 (8.1)
**Acitetrin**, n (%)	0	3 (1.7)	1 (0.6)	0	1 (1.4)	0
**CCS**, n (%)	0	1 (0.6)	1 (0.6)	0	0	0
**NSAIDs**, n (%)	0	0	0	0	0	0
**PUVA**, n (%)	0	0	1 (0.6)	0	0	0
**Apremilast**, n (%)	1(2.1)	0	2 (1.2)	0	1 (1.4)	0
**Switched**, n (%)	25 (52.1)	50 (28.6)	23 (13.8)	1 (8.3)	4 (5.5)	0
*Adverse events*						
**AEs**, n (%)	9 (18.7)	28 (16)	30 (17.9)	4 (33.3)	11 (15.1)	0
**SAEs**, n (%)	0	3 (1.7)	2 (1.2)	0	0	0
**AEs onset after treatment initiation,** months^1^	8.2 ± 4.3	11.2 ± 6.1	10.3 ± 5.0	7.7 ± 3.5	5.4± 4.8	-

IFX, infliximab; ETN, etanercept; ADA, adalimumab; GOL, golimumab UST, ustekinumab; SEC, secukinumab; MTX, methotrexate; CyA, cyclosporin A; CCS, corticosteroids; NSAIDs, Nonsteroidal anti-inflammatory drugs; PUVA, Psoralen Ultra-Violet A; AEs, adverse events; SAEs, serious adverse events.

^1^Excluding immediate administration site reactions and allergic reactions to excipient.

Moreover, 104 patients (20.3%) received concomitant treatment with one or more immunomodulatory drugs, *Psoralen Ultra-Violet A* (PUVA) or corticosteroids (CCS). No one received chronically non-steroidal anti-inflammatory drug (NSAIDs).

At the index date, most patients (394; 77.0%) were naïve to biologic treatment, whereas the remaining switched/swapped from one or more previous biologic drugs (number of previous biologic drugs range 1–3). Data reported in Table 3 refer to switches occurred during study period for AEs or treatment ineffectiveness.

**Table 3:** Details on switches between biologic drugs.

	*Switch to*						
*Switch from*		**IFX**	**ETN**	**ADA**	**GOL**	**UST**	**SEC**
	**IFX**		4	7 (4)	5	5 (1)	6
	**ETN**	9		15	7 (1)	14 (1)	3
	**ADA**	4	4 (3)		1	12 (2)	3
	**GOL**	/	/	1		/	/
	**UST**	/	/	1	/		3
	**SEC**	/	/	/	/	/	

Switches related to inefficacy (switches related to AEs); IFX, infliximab; ETN, etanercept; ADA, adalimumab; GOL, golimumab, UST, ustekinumab; SEC, secukinumab.

Overall, 376 patients (73.4%) have not developed AEs or therapeutic failures, whereas 82 patients (16.1%) experienced at least one AE and 59 (11.6%) had at least a primary/secondary failure. Differences of these three groups are summarized in Table 4. In detail, comparing a specific treatment (*i*.*e*. a specific biologic drug) with the remaining cohort, treatment with infliximab and etanercept were mostly associated with primary/secondary failures (*p* < 0.001 and *p* = 0.004). On the other hand, ustekinumab seems to be associated with a low rate of AEs (p = 0.01) and no adverse events (p = 0.04) or failures (p = 0.02) have been reported with secukinumab treatment. No statistical difference was noticed with other variables, and none, excluding secukinumab, was associated with AEs insurgence.

**Table 4:** Clinical and demographic features compared among sub-groups.

	Patients without AEs or failures N. 376 (%)	Patients with AEs N. 82(%)	*p* value#	Patients with failures N. 59 (%)	*p* value¶
**Sex**			0.57		0.89
Females	147 (39.1)	35 (42.7)		24 (40.7)	
Males	229 (60.9)	47 (57.3)		35 (59.3)	
**Mean age (±SD)**	54.8 ±12.9	55.1 ±13.7	0.91	54.9 ±15.6	0.76
**Mean age at first administration(±SD)**	51.5 ±13.4	52.2 ±13.9	0.50	49.9 ±16.2	0.29
**Diagnosis**			0.13		0.79
Plaque psoriasis	216 (57.4)	39 (47.6)		33 (55.9)	
Psoriatic arthritis	160 (42.6)	43 (52.4)		26 (44.1)	
**Biologic drugs**					
**Naive**	292 (77.7)	66 (80.5)	0.41	40 (67.8)	0.07
**Biologic treatment**					
Infliximab	27 (7.2)	9 (11.0)	0.58	14 (23.7)	0.001*
Etanercept	120 (31.9)	28 (34.1)	0.99	30 (50.8)	0.004*
Adalimumab	124 (33.0)	30 (36.6)	0.40	13 (22.0)	0.65
Golimumab	8 (1.9)	4 (4.9)	0.09	0	**0.60**
Ustekinumab	60 (16.0)	11 (13.4)	0.71	2 (3.4)	0.01*
Secukinumab	37 (9.8)	0	0.04*	0	0.02*
**Non biological concomitant therapy**					
Methotrexate	39 (10.4)	6 (7.3)	0.35	8 (13.6)	0.36
Cyclosporine	59 (15.7)	7 (8.5)	0.08	12 (20.3)	0.21
Acitetrin	4 (1.1)	0	-	1 (1.7)	0.55
Corticosteroid (CCS)	2 (0.5)	0	-	0	-
PUVA	1 (0.3)	0	-	0	-
Apremilast	3 (0.8)	0	-	1 (1.7)	0.40

*Statistically significant.

#Patients without AEs or failures versus patients with AEs.

¶Patients without AEs or failures versus patients with failures.

PUVA, Psoralen Ultra-Violet A; AEs, adverse event.

Furthermore, the relationship between treatment and AEs insurgence or failures have been assessed performing univariate and multivariate analysis. Indeed, according to regressions, the probability of experiencing treatment failure was significantly higher in patients treated with infliximab (OR 3.56; CI 95% 1.75–7.23) and etanercept (OR 2.20; CI95% 1.26–3.81), whereas lower with ustekinumab (OR 0.19; CI95% 0.05–0.82) (Table 5).

**Table 5:** Univariate and multivariate regressions on adverse events insurgence and treatment failures.

	Adverse events		Primary/secondary failure	
	Univariate (95%CI)	Multivariate (95%CI)^1^	Univariate (95%CI)	Multivariate (95%CI)^1^
**Infliximab**	1.24 (0.57–2.67)	1.42 (0.65–3.12)	3.83 (1.91–7.68)	3.56 (1.75–7.23)
**Etanercept**	0.99 (0.61–1.64)	1.01 (0.61–1.67)	2.19 (1.27–3.79)	2.20 (1.26–3.81)
**Adalimumab**	0.81 (0.49–1.33)	1.21 (0.73–1.98)	0.55 (0.29–1.05)	0.54 (0.28–1.05)
**Golimumab**	2.70 (0.79–9.20)	3.03 (0.86–10.51)	-	-
**Ustekinumab**	0.92 (0.46–1.83)	0.87 (0.43–1.74)	0.18 (0.04–0.79)	0.19 (0.05–0.82)

^1^Adjusted for age, sex, concomitant drugs, and treatment duration.

^2^No AEs or failures have been reported with ustekinumab.

### Characteristics of adverse events

During the study period, we reported 82 (16.1%) patients experiencing at least one AEs and 5 (1.0%) SAEs among them, for a total of 118 adverse events. %). Naranjo probability scale documented a probable association (Naranjo Score value 6 to 8) for all AEs detected. However, AEs/SAEs have been reported mainly for patients in treatment with GOL (4/12; 33.4%), and in descending order of prevalence for IFX (9/48; 18.7%), ADA (32/167; 19.2%), ETN (31/175; 17.7%) and UST (11/73; 15.1%). No adverse events have been observed with SEC (0/37). The most common adverse events were injection site reactions and skin disorders and all the AEs observed were expected (namely already reported in summary of product characteristic). An exhaustive list of AEs and SAEs categorized according to the MedDRA dictionary is provided in S1 Table.

On overall population, only five patients (1%) experiencing SAEs were reported during the study period: one case of severe pneumonia (infliximab; 2.1% of IFX treated patients), three cases with etanercept (1.7%) in particular: one case of benign respiratory tract neoplasm, one case of new-onset lupus-like syndrome and a haemorrhagic cystitis. Finally, a case of severe splenomegaly leading to hospitalization was reported with adalimumab (0.5%).

Finally, we have questioned the RNF to assess the reporting ratio of AEs with biologic therapy to treat psoriasis from the same tertiary centres during the previous two years of our program. Overall, only 7 AEs have been previously reported, in particular: 5 with ADA, one with IFX and one with ETN and no SAE. However, considering the lack of patients treated in the same period and details of prescriptions, an AEs’ ratio can not be calculated.

## Discussion

To date, several biologics have been approved for the treatment of psoriasis and psoriatic arthritis, and further are in development. Other than effectiveness, safety is crucial for patients and physicians, directly influencing adherence to treatment and quality of life.

Spontaneous reporting of suspected AEs, and above all programs of active pharmacovigilance, can provide a rapid and early detection of adverse events potentially related to drugs but a high under reporting rate, with an overall estimate of only 6–10% of all AEs described, is widespread [9,15].

Our findings show that more than 16.1% of the patients (treated mostly with etanercept and secondarily with adalimumab) incurred AEs. Overall, only 4.2% AEs were serious, as required hospitalization, or consisted of clinically relevant conditions.

Approximately the same percentage of patients experiencing adverse events (range 15.1–19.2%) have been reported with each biologic drug, except for GOL (33%). However, the very low number of patients in treatment with GOL in our cohort (12/512; 2.3%) can not allow further considerations and analysis.

In agreement to literature [16,17], the most common AEs observed in our study belong to the general disorders and administration site conditions (mild to moderate), followed by investigations anomalies. Increased rates of infection have been reported in patients receiving TNF-α inhibitors, with upper respiratory tract infections, pharyngitis and sinusitis, most commonly reported [16], as well as an increased risk of opportunistic infections [18].

We have reported only 11 not-severe cases of infections, in particular three candidiasis and four herpes simplex infections, two cases of pneumonia (of which one severe leading to hospitalization and drug discontinuation) and four rhino-pharyngitis, all in patients not in concomitant corticosteroids usage and only with TNF-α inhibitors (with the exception of one case of pharyngitis). No rare opportunistic infections (*e*.*g*. *pneumocystis jirovecii*, *histoplasma capsulatum*, *listeria monocytogenes*) or tuberculosis reactivation have been described.

Some post-marketing reports [19,20] have described several hepatic reactions, hepatitis and acute liver failures in patients treated with TNF-α inhibitors (mainly with infliximab), even though the risk is very slight and casual relationship has not established. We have reported 9 cases of increased transaminases during treatment with etanercept and adalimumab, leading only to one case of discontinuation with etanercept (transaminases 3-fold higher and normalized with withdraw).

As before mentioned, five SAEs have been reported including a lupus-like syndrome, an AE rarely reported in literature with TNFα-inhibitors treatment [21], resolved after etanercept discontinuation, and a benign respiratory tract neoplasm, although Kimball and colleagues [22] demonstrated that neoplastic risk was higher in psoriatic patients but likely not related with biologic treatments.

The time to drug discontinuation is influenced by several factors such as loss or lack of efficacy, adverse events and poor adherence, among others [23]. However, the causes for primary and secondary failure of TNF antagonists, defined as no responses to a new TNF inhibitor administered or fails to respond within 16 weeks, are still not completely understood.

Primary or secondary failure occurred in 59 (11.5%) patients and were significantly correlated with the use of infliximab and etanercept. Our data are in agreement to a recent meta-analysis [24] of real world studies regarding etanercept, highly associated with discontinuation due to loss of efficacy, and ustekinumab, reporting a low rate of discontinuation. On the other hand, infliximab is not commonly discontinued for loss of efficacy, in contrast to our findings. However, the small sample size and patients’ baseline characteristics as well as co-treatments could have influenced drug discontinuation.

The best safety and effectiveness profiles have been reported in our cohort with secukinumab, considering the absence of AEs (p = 0.04) and failures (p = 0.02) reported during follow-up. However, these results should be evaluated considering that patients treated with secukinumab have a milder disease compared with other patients (*i*.*e*. 5% with psoriatic arthritis) and the descriptive design limit additional considerations.

Regarding the ratio of AEs reports related to biologic treatments in our region, a considerable improvement has been achieved in the last four years due to active pharmacovigilance programs, as demonstrated in our previous study in rheumatology [9] and gastroenterology [13] and generally such as reported in literature [25,26]. In this study, the number of AEs reported seems to be considerably increased (from 7 to 87) compared to the previous two years in the same centres, although the overall number of patients treated subdivided per drug are mandatory to assess confounding variables and confirm improvement. However, considering the higher AEs ratio described in dermatology registries as the BAD Biologic Interventions Register (BADBIR) [27] or the Spanish Registry of Adverse Events Associated with Biologic Drugs in Dermatology (BIOBADADERM) [28], our AEs reports need to be further implemented and long term follow-up need to be planned. Our data must be considered according to some limitations: the most evident is the small sample size *per* drug that has limited the possibility to detect differences among treatments regarding switch or to analyse all the confounder factors that could influence AEs insurgence. Furthermore, our follow up period is limited, and some variables have been not reported in our database.

## Conclusion

Biological agents are highly targeted and effective therapies, that allowed to move forward the Psoriasis Area Severity Index (PASI) from 75 to 90/100 as a primary endpoint measurement in clinical trials, with the aim not only to improve but to clear psoriasis [6], changing completely its management.

Even though biased by the limitations mentioned above as the small sample size and the limited follow-up period, our study provides useful data on widely used biologic drugs and their tolerability, discontinuation rate and the incurrence of severe adverse events. Further studies are necessary to include the new approved biologics for psoriasis, improve the sample size and plan long-term follow-up.

## Supporting information

S1 Table MedDRA-compliant description of adverse events (AEs). *Classified as serious adverse event (SAE). *IFX*, *infliximab; ETN*, *etanercept; ADA*, *adalimumab; GOL*, *golimumab*, *UST*, *ustekinumab; SEC*, *secukinumab; SOC*, *system organ class; PT*, *preferred term*.
(DOCX)
