# Supplementary material for: Safety profile of biologic drugs for psoriasis in clinical practice: An Italian prospective pharmacovigilance study
Source: PLoS One. 2020 Nov 3;15(11):e0241575. doi: 10.1371/journal.pone.0241575 (PMC7608898; doi:10.1371/journal.pone.0241575)
Supplement: S1 Table — *Classified as serious adverse event (SAE). IFX, infliximab; ETN, etanercept; ADA, adalimumab; GOL, golimumab, UST, ustekinumab; SEC, secukinumab; SOC, system organ class; PT, preferred term. (DOCX) [file pone.0241575.s001.docx]

| **Table S1.** MedDRA-compliant description of adverse events (AEs) | | | | | | | |
| --- | --- | --- | --- | --- | --- | --- | --- |
|  | **IFX** | **ETN** | **ADA** | **GOL** | **UST** | **SEC** | **Total** |
| **SOC - General disorders and administration site conditions** | 2 | 15 | 17 | 4 | 3 |  | 41 |
| PT - Hyperhidrosis |  | 1 |  |  |  |  | 1 |
| PT1 - Asthenia | 1 | 9 | 9 | 2 | 3 |  | 24 |
| PT2 - Hot flush |  | 1 |  |  |  |  | 1 |
| PT3 - Pallor |  | 1 |  |  |  |  | 1 |
| PT4 - Administration site reactions | 1 | 3 | 7 | 1 |  |  | 12 |
| PT5 - Peripheral oedema |  |  | 1 | 1 |  |  | 2 |
|  |  |  |  |  |  |  |  |
| **SOC - Vascular disorders** |  | 1 | 1 |  |  |  | 2 |
| PT - Cyanosis |  |  | 1 |  |  |  | 1 |
| PT1 - Hypotension |  | 1 |  |  |  |  | 1 |
|  |  |  |  |  |  |  |  |
| **SOC - Skin and subcutaneous tissue disorders** | 2 | 1 | 2 |  |  |  | 5 |
| PT - Folliculitis | 1 |  |  |  |  |  | 1 |
| PT1 - Rash | 1 |  |  |  |  |  | 1 |
| PT2 - Pruritus |  | 1 | 2 |  |  |  | 3 |
|  |  |  |  |  |  |  |  |
| **SOC - Ear and labyrinth disorders** |  | 1 | 2 |  | 1 |  | 4 |
| PT - Vertigo |  | 1 | 2 |  | 1 |  | 4 |
|  |  |  |  |  |  |  |  |
| **SOC - Nervous system disorders** |  | 1 | 3 |  | 1 |  | 5 |
| PT - Headache |  | 1 | 3 |  | 1 |  | 5 |
|  |  |  |  |  |  |  |  |
| **SOC - Infections and infestations** | 1 | 2 | 3 |  | 1 |  | 7 |
| PT - Candidiasis infection |  |  | 2 |  | 1 |  | 3 |
| PT1 - Herpes virus infection | 1 | 2 | 1 |  |  |  | 4 |
|  |  |  |  |  |  |  |  |
| **SOC - Respiratory, thoracic and mediastinal disorders** | 1 | 4 | 5 |  | 2 |  | 12 |
| PT - Pneumonia | 1* | 0 | 1 |  |  |  | 2 |
| PT1 - Interstitial pneumonia |  | 1 | 1 |  |  |  | 2 |
| PT2 - Nasopharyngitis |  | 1 | 2 |  | 1 |  | 4 |
| PT3 - Benign respiratory tract neoplasm |  | 1* |  |  |  |  | 1 |
| PT4 - Dyspnoea |  | 1 | 1 |  | 1 |  | 3 |
|  |  |  |  |  |  |  |  |
| **SOC - Investigations** |  | 9 | 4 | 1 | 3 |  | 17 |
| PT - Transaminases increased |  | 6 | 1 |  | 1 |  | 8 |
| PT1 - Hepatitis C antibody positive |  | 1 |  |  |  |  | 1 |
| PT2 - Carcinoembryonic antigen increased |  | 2 | 1 |  | 1 |  | 4 |
| PT3 - Blood count abnormal |  |  |  | 1 |  |  | 1 |
| PT4 - Haemoglobin decreased |  |  | 1 |  | 1 |  | 2 |
| PT5 - Red blood cell sedimentation rate abnormal |  |  | 1 |  |  |  | 1 |
|  |  |  |  |  |  |  |  |
| **SOC - Blood and lymphatic system disorders** |  | 4 | 1 |  | 2 |  | 7 |
| PT - Lymphocytosis |  |  |  |  | 1 |  | 1 |
| PT1 - Thrombocytopenia |  | 3 |  |  |  |  | 3 |
| PT2 - Anaemia |  | 1 |  |  | 1 |  | 2 |
| PT3 - Splenomegaly |  |  | 1* |  |  |  | 1 |
|  |  |  |  |  |  |  |  |
| **SOC - Gastrointestinal disorders** |  | 2 | 8 |  |  |  | 10 |
| PT - Vomiting |  |  | 2 |  |  |  | 2 |
| PT1 - Gingivitis |  | 1 |  |  |  |  | 1 |
| PT2 - Nausea |  | 1 | 4 |  |  |  | 5 |
| PT3 - Diarrhoea infectious |  |  | 2 |  |  |  | 2 |
|  |  |  |  |  |  |  |  |
| **SOC - Immune system disorders** | 5 | 1 |  |  |  |  | 6 |
| PT - Allergic reaction to excipient | 5 |  |  |  |  |  | 5 |
| PT1 - Lupus-like syndrome |  | 1* |  |  |  |  | 1 |
|  |  |  |  |  |  |  |  |
| **SOC - Renal and urinary disorders** |  | 1 |  |  | 1 |  | 2 |
| PT - Haematuria |  |  |  |  | 1 |  | 1 |
| PT1 - Haemorrhagic Cystitis |  | 1* |  |  |  |  | 1 |
|  |  |  |  |  |  |  |  |
| **Classified as serious adverse event (SAE)*  *IFX, infliximab; ETN, etanercept; ADA, adalimumab; GOL, golimumab, UST, ustekinumab; SEC, secukinumab; SOC,*  *system organ class; PT, preferred term* | | | | | | | |
